# Incidence and risk factors of severe adverse events with nevirapine-based antiretroviral therapy in HIV-infected women. MTCT-Plus program, Abidjan, Côte d'Ivoire

**DOI:** 10.1186/1471-2334-10-188

**Published:** 2010-06-24

**Authors:** Patrick A Coffie, Besigin Tonwe-Gold, Aristophane K Tanon, Clarisse Amani-Bosse, Gédéon Bédikou, Elaine J Abrams, François Dabis, Didier K Ekouevi

**Affiliations:** 1 Programme MTCT-Plus, ACONDA, BP: 1954 Abidjan 18, Abidjan, Côte d'Ivoire; 2 Institut de Santé Publique, Epidémiologie et Développement (ISPED), Université Victor Segalen Bordeaux 2, 149 rue Leo Saignat, 33076, Bordeaux, France; 3 Centre de Recherche INSERM U897, 149 rue Leo Saignat, 33076, Bordeaux, France; 4 Service des Maladies Infectieuses et Tropicales, Centre Hospitalier Universitaire Treichville, BP: 1954 Abidjan 18, Abidjan, Côte d'Ivoire; 5 MTCT-Plus Initiative, International Center for AIDS Care and Treatment Programs (ICAP), Mailman School of Public Health, Columbia University, 722 West 168th Street, 7th floor, New York, NY, 10032, USA New-York, NY, USA

## Abstract

**Background:**

In resource-limited settings where nevirapine-containing regimen is the preferred regimen in women, data on severe adverse events (SAEs) according to CD4 cell count are limited. We estimated the incidence of SAEs according to CD4 cell count and identify their risk factors in nevirapine-treated women.

**Methods:**

All HIV-infected women who initiated nevirapine-containing regimen in the MTCT-Plus operational program in Abidjan, Côte d'Ivoire, were eligible for this study. Laboratory and clinical (rash) SAEs were classified as grade 3 and 4. Cox models were used to identify factors associated with the occurrence of SAEs.

**Results:**

From August 2003 to October 2006, 290 women initiated a nevirapine-containing regimen at a median CD4 cell count of 186 cells/mm^3 ^(IQR 124-266). During a median follow-up on treatment of 25 months, the incidence of all SAEs was 19.5/100 patient-years. The 24-month probability of occurrence of hepatotoxicity or rash was not different between women with a CD4 cell count >250 cells/mm^3 ^and women with a CD4 cell count ≤250 cells/mm^3 ^(8.3% *vs*. 9.9%, Log-rank test: p = 0.75). In a multivariate proportional hazard model, neither CD4 cell count >250 cells/mm^3 ^at treatment initiation nor initiation NVP-based regimen initiated during pregnancy were associated with the occurrence of SAEs.

**Conclusion:**

CD4 cell count >250 cells/mm^3 ^was not associated with a higher risk of severe hepatotoxicity and/or rash, as well as initiation of ART during pregnancy. Pharmacovogilance data as well as meta-analysis on women receiving NVP in these settings are needed for better information about NVP toxicity.

## Background

At the end of 2007, more than three million people were receiving Highly Active Antiretroviral Therapy (HAART) in resource-limited settings [1]. The first-line regimen recommended by the World Health Organization (WHO) in resource-limited includes two nucleoside reverse transcriptase inhibitors (NRTI) with one non-nucleoside reverse transcriptase inhibitor (NNRTI) [2].

Nevirapine (NVP) is the preferred NNRTI in first-line antiretroviral regimens in pregnancy because of substantial clinical experience with pregnant women and its proven efficacy in reducing mother-to-child transmission [3,4]. The most frequent adverse events of NVP are hepatotoxicity and cutaneous rash. Studies from developed countries report an increased risk of severe hepatotoxicity and cutaneous rash in women with CD4 count >250 cells/mm^3 ^[5-9]. Based on these findings, WHO recommends avoiding NVP in women, including pregnant women, with CD4 counts between 250 and 350 cells/mm^3^, or if no other options exist as it is often the case in resource-limited settings, using it with caution and close monitoring during the first 12 weeks of therapy [2,8]. Although some cases have been reported in pregnant women, it is still not known whether pregnancy increases the risk of the occurrence of hepatotoxicity or rash [9-11]. In resource-limited settings, where NVP is widely used in women and during pregnancy whatever the level of CD4, there is little data on the occurence of hepatotoxicity and/or rash according to CD4, and all except one study did not find this association [12-16].

We hypothesize that HAART initiation among pregnant HIV-infected women or HIV-infected women with CD4 count >250 cells/mm^3 ^may be associated with an increased frequency of SAEs. Thus, we estimated in the MTCT-Plus program in Abidjan, Côte d'Ivoire, the incidence rate of all severe adverse events (SAEs) according to CD4 cell count and pregnancy status, especially hepatotoxicity and/or rash and investigated their risk factors in women initiating NVP-based HAART.

## Methods

### Study design and setting

A prospective cohort study was conducted in Abidjan, Côte d'Ivoire between August 2003 and October 2006 among women enrolled in the MTCT-Plus program, which is built upon existing prevention of mother-to-child transmission (PMTCT) services and provides HIV-infected women, their partners, and their children, holistic family care with unrestricted access to HAART for eligible patients [17,18].

### Study population

All HIV-infected women were included in this study if they initiated NVP-based HAART according to the following eligibility criteria: WHO clinical stage 2 (until December 2004) or stage 3 and CD4+ T lymphocytes (CD4 cell count) <350 cells/mm^3^, or stage 4 regardless of CD4 cell count, or CD4 cell count <200 cells/mm^3^. The women had to meet one additional biological criterion before initiating HAART: aminotransferase and/or alaninetransferase levels no more than three times the upper limit of the normal range. All women with a CD4 cell count <350 cells/mm^3 ^were also prescribed co-trimoxazole prophylaxis. All women were ARV-naive with the exception of prior PMTCT exposure.

### Ethical aspects

The MTCT-Plus program was reviewed by the institutional review board (IRB) from Columbia University in 2000 (principal sponsor) and was not considered a research project but rather a demonstration program in the context of the ARV roll-out. As an operational HIV care and treatment program MTCT-Plus program was exempted from review by the IRB from the Ministry of health of Côte d'Ivoire.

### Enrollment and follow-up

Maternal socio-demographic, clinical and biological characteristics were recorded at enrollment in the program and at initiation of HAART [18]. During weekly follow-up visits for the first two months and monthly visits thereafter, clinical signs and symptoms, drug intake, and tolerance were assessed. Between scheduled visits, women had free access to the study clinics for medical problems. Total blood cell count (MaxM, BeckmanCoulter, Miami, FL, USA), serum liver enzymes, and serum creatinine were monitored according to the following schedules: in women starting HAART after delivery at baseline, month 1, and every three months thereafter; in women starting HAART prepartum at baseline, week 2, month 1, and monthly thereafter during pregnancy. CD4 cell count were measured using a dual-platform flow cytometry technique with an automated blood cell counter (MaxM, BeckmanCoulter, Miami, FL, USA), at the screening visit and then every six months. Serology for hepatitis B was not routinely performed. All blood samples were processed in the same laboratory.

### Outcomes and definitions

The outcomes of interest were the occurrence of SAEs defined as clinical and biological grade ¾ adverse events according to the Agence Nationale de la Recherche sur le Sida et les hepatites virales (ANRS) table for grading the severity of adult adverse events [19]: at least one haemoglobinaemia measurement <70 g/l, one neutrophil count <750/mm^3^, one amino and/or alaninetransferase measurement more than five times the upper limit of the normal range and any extended papulovesicular or oozing eruption, palpable purpura (suggestive of vasculitis), polymorphous erythaema, small-size cutaneous or mucous ulcerations, any blistering cutaneous and/or mucosal lesions (Lyell or Stevens-Johnson), febrile erythrodermia, whether or not associated with other signs indicative of hypersensitivity, cutaneous necrosis requiring surgical excision. All SAEs were reported by clinicians during follow-up visits and subsequently validated by two expert clinicians. If the experts disagree, a third expert was called upon to avoid misclassification and to validate a final diagnosis.

### Statistical analysis

Group comparisons used Student's t-test, non-parametric Mann-Whitney U test or variance analysis for continuous variables, and the Chi^2 ^test or Fisher's exact test for categorical variables. The incidence rate of SAEs per 100 patient-months or years was estimated with its 95% confidence interval (CI). The Log-Rank test was used to compare the incidence rate between two groups. Univariable and then multivariable Cox regression analyses were performed to identify factors associated with the occurrence of these SAEs. All factors associated with the outcomes at a P value <0.25 were included in the multivariable analysis. The CD4 cell count and WHO clinical stage at HAART initiation were kept in the final model. Adjusted hazard ratios (aHR) and their 95% CI are reported with two-sided p-values. All analyses were performed in intent-to-treat and on treatment [20] with SAS software version 9.1 (SAS Institute, Cary, NC, USA).

## Results

### Patients and follow-up

From August 2003 to October 2006, 530 women were enrolled in MTCT-Plus and 290 (54.7%) women initiated a NVP-containing regimen and were included in this study. Their median age at HAART initiation was 29 years (inter-quartile range [IQR] 26-33) and median CD4 cell count was 186 cells/mm^3 ^(IQR 124-266). Two-hundred and two women (70%) initiated HAART with a CD4 cell count ≤250 cells/mm^3^, 130 (44.8%) were at WHO clinical stage 3 or 4, 125 (43.0%) initiated HAART during pregnancy, and 287 (99.0%) started co-trimoxazole. During a median follow-up on HAART of 25 months (IQR 14-30), seven women (2.4%) were lost to follow-up, seven (2.4%) stopped HAART on their own initiative and 16 (5.5%) died. The baseline and follow-up characteristics of these women are summarized in Table 1.

**Table 1:** Baseline and follow-up characteristics of HIV-infected women in the MTCT-Plus program (N = 290).

At initiation of treatment	
Period at HAART initiation	
Pregnant/non pregnant, n (%)	125/165 (43/57)
Exposed women to PMTCT	
Exposed/no exposed, n (%)	153/137 (53/47)
Age, years, median [IQR]	29 [26-33]
>29 years	137 (47)
Body mass index, Kg/m^2 ^[IQR]	22.3 [20.1-25.3]
>18.5 *	250 (86)
WHO clinical stage, n (%)	
1	33 (11)
2	127 (44)
3	117 (40)
4	13 (5)
CD4 counts, cells/mm^3 ^[IQR]	186 [124-266]
>250	88 (30)
Co-trimoxazole, n (%)	287 (99)
Haemoglobin level, g/l, median [IQR]	9.8 [9-11]
≤9.8	150 (52)
Neutrophil count, mm^3^, median [IQR]	2639 2639 [1911-3712]
<1500	28 (10)
HAART regimen, n (%)	
ZDV/3TC/NVP	265 (91)
d4T/3TC/NVP	25 (9)
Alanine aminotransferase, UI, median [IQR]	15 [11-24]
<31 IU/L*	240 (83)
Aspartate aminotransférase, UI, median [IQR]	24 [19-31]
<32 IU/L*	235 (81)

**Follow-up **	

Cumulative, person-months	6388
Per patient, months, median [IQR]	25 [14-30]
Status on study termination	
Dead, n (%)	16 (6)
Lost to follow-up, n (%)	7 (2)
Alive, n (%)	267 (92)

HAART: Highly active antiretroviral therapy; PMTCT: prevention to mother-to-child transmission; WHO: World Health Organization; IQR: interquartile range; ZDV: zidovudine; 3TC: lamivudine; NVP: nevirapine; d4T: stavudine; ALT: alanine aminotransferase; AST: aspartate aminotransferase; IU: International unity;

* upper limit of normal

### Antiretroviral therapy

The first-line HAART regimen was ZDV/3TC/NVP in 265 women (91.4%) and stavudine (d4T)/3TC/NVP in 25 women (8.6%). In this cohort, 153 women (52.8%) were previously exposed to a PMTCT regimen during a previous pregnancy: short-course (sc) ZDV and lamivudine plus single dose nevirapine (sdNVP) (n = 84), scZDV plus sdNVP (n = 65) and scZDV alone (n = 2). The median interval between exposure to PMTCT and initiation of HAART was 22 months (IQR 13-21).

### Incidence rate of severe adverse events

A total of 104 SAEs were reported in 88 women (30.3%) as follows: neutropenia (n = 60; 20.7%), anaemia (n = 17; 5.9%), rash (n = 15; 5.2%), hepatotoxicity (n = 10; 3.4%), headache and neuropathy (n = 1; 0.3%).

**Figure 1:**
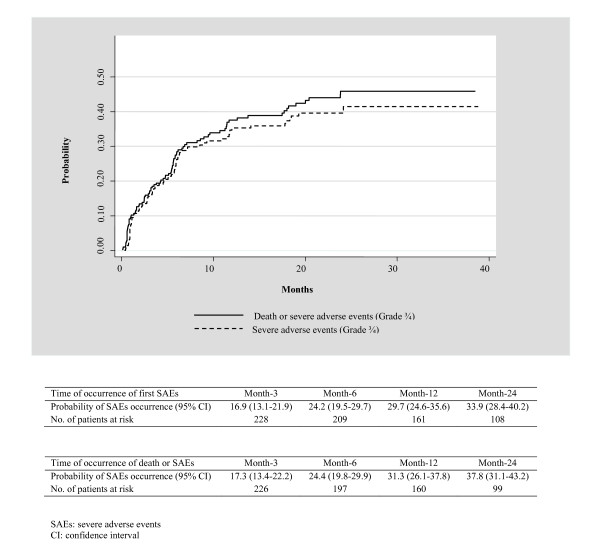
Probability of occurrence of severe adverse events (grade ¾) and/or death in women initiating a nevirapine-based antiretroviral therapy in the MTCT-Plus program in Abidjan, Côte d'Ivoire

The overall incidence rate of SAEs was 19.5/100 patient-years (PY), (95% CI 15.9-23.4): 13.3/100 PY (95% CI 10.3-16.8) for neutropenia, 3.8/100 PY (95% CI 2.2-6.0) for anemia, 3.3/100 PY (95% CI 1.9-5.4) for rash, and 2.2/100 PY (95% CI 1.1-4.0) for hepatotoxicity. The median delay between HAART initiation and occurrence of all SAEs was 3.0 months (IQR 1-6). It was 6.0 months (IQR 2.5-7.5) for neutropenia, 4.0 months (IQR 2-5) for anemia, 2.5 months (IQR 1-11) for hepatotoxicity, and 1.0 month (IQR 1-3) for rash. The overall probability of occurrence of SAEs was 17.0% (95% CI 13.1-21.9%) at month-3, 29.7% (95% CI 24.6-35.6%) at month-12, 33.9% (95% CI 28.4-40.2%) at month-24 (Figure 1).

Among the 16 women who died, eight (50.0%) had a history of SAEs. Table 2 describes the death cases. When considering death or SAEs, the 12-month and 24-month were 32.1% (95% CI 26.8-38.0%) and 36.6% (95% CI 30.9-42.9%) respectively (Figure 1).

**Table 2:** Causes of death among HIV-infected women followed in the MTCT-Plus program and treated with NVP-based HAART.

#	CD4 count* cells/mm ^**3**^	Pregnancy at HAART initiation	Regimen*	Cause of death	Time of death** (months)	History of SAEs	Description of SAEs	Time of occurrence of SAEs** (months)
1	253	No	AZT/3TC/NVP	Mulitple myeloma	26.7	Yes	Neutropenia grade 3	7.2
2	241	No	AZT/3TC/NVP	Hepatitis B	24.6	Yes	Hepatotoxicity grade 3	24.2
3	332	No	AZT/3TC/NVP	Hepatitis B	13.3	Yes	Anaemia grade 4	1.2
4	230	No	AZT/3TC/NVP	Unknown	24.9	Yes	Neutropenia grade 4	9.2
5	271	Yes	AZT/3TC/NVP	Severe anaemia	3.7	Yes	Anaemia grade 4	3.4
6	250	No	AZT/3TC/NVP	AIDS terminal	14.0	Yes	Neutropenia Grade3	4.6
7	193	No	AZT/3TC/NVP	Cerebral malaria	20.8	Yes	Rash grade 3	0.7
8	39	Yes	AZT/3TC/NVP	AIDS terminal	10.6	Yes	Rash grade 3	2.0

9	170	Yes	AZT/3TC/NVP	AIDS terminal	20.4	No		
10	186	Yes	d4T/3TC/NVP	Eclampsia	1.8	No		
11	7	No	AZT/3TC/NVP	Pneumonia	6.9	No		
12	226	No	d4T/3TC/NVP	Renal tumor	2.0	No		
13	121	No	AZT/3TC/NVP	Cerebral malaria	10.0	No		
14	84	Yes	AZT/3TC/NVP	Fever	9.8	No		
15	24	No	AZT/3TC/NVP	Meningitis	7.4	No		
16	288	Yes	AZT/3TC/NVP	Gastric perforation	12.9	No		

* At initiation of treatment, SAEs = Severe adverse events grade III/IV

** After HAART initiation

### Severe adverse events and CD4 count at the initiation of treatment

The median CD4 cell count was 151 cells/mm^3 ^(IQR 106-190) for women who initiated HAART with a CD4 cell count ≤250 cells/mm^3 ^and 303 cells/mm^3 ^(IQR 275-331) for those who initiated HAART at >250 cells/mm^3^. Baseline characteristics such as age, body mass index (BMI), and WHO clinical stage were comparable between the two groups except for the proportion of women who initiated HAART during pregnancy: in women with a CD4 cell count ≤250 cells/mm^3^, 47% initiated HAART while pregnant *vs. *34% in women with a CD4 cell count >250 cells/mm^3 ^(p = 0.04). The 24-month probability of occurrence of rash or hepatotoxicity was not different between women with a CD4 cell count >250 cells/mm^3 ^and women with a CD4 cell count ≤250 cells/mm^3 ^(8.3% *vs*. 9.9%, Log-rank test: p = 0.75). Similarly, this 24-month probability was not different between the two groups when considering death and SAEs combined (27.0% *vs*. 40.7% respectively, Log-rank test, p = 0.08).

### Severe adverse events and period of HAART initiation

**Figure 2:**
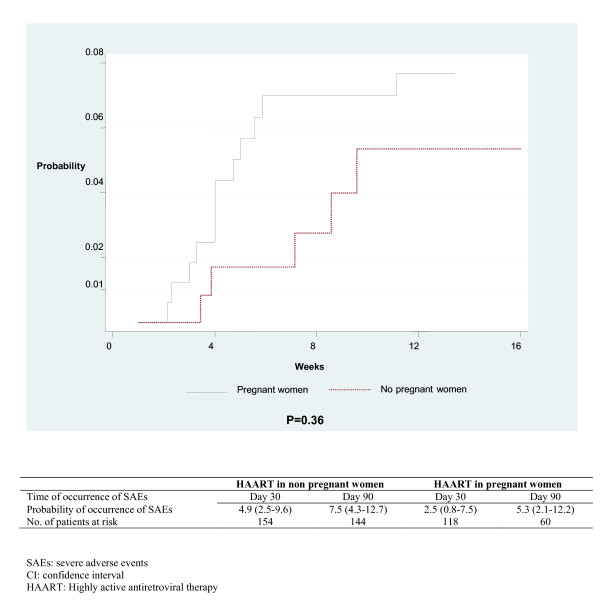
**Probability of occurrence of grade III/IV rash or hepatotoxicity during the three months of follow-up in women initiating a nevirapine-based antiretroviral therapy according to the period of the initiation of treatment (pregnant and non pregnant women) **. MTCT-Plus program in Abidjan, Côte d'Ivoire

The baseline characteristics such as age, BMI, WHO clinical stage and CD4 cell count, were comparable between the women who initiated HAART during pregnancy (group 1) and those who did not (group 2). The median interval between HAART initiation and delivery was 3.0 months (IQR 2-3) in women in group 1 and during this period, the incidence rate was 3.4/100 patient-months (IQR 1.7-6.1) for all SAEs and 1.6/100 patient-months (IQR 0.5-3.6) for rash and/or hepatotoxicity. In women in group 2, when restricting the analyses to the first three months of follow-up after HAART initiation, the incidence rate was 6.1/100 patient-months [IQR 4.1-8.6] for all SAE and 2.5/100 patient-months [IQR 1.3-4.3] for rash and/or hepatotoxicity. The 3-month probability of occurrence of rash or hepatotoxicity did not differ between groups 1 and 2 (5.3% *vs. *7.5%; Log-Rank test, p = 0.35) (Figure 2).

### Evolution of severe adverse events

One hundred and four SAEs were reported and led to a change in treatment in 47 cases. NVP was switched to a protease inhibitor secondary to grade 3 rash (n = 15) or grade 3/4 liver toxicity (n = 4), or to EFV (n = 3) or abacavir (n = 1) owing to grade 3/4 liver toxicity. For two cases of hepatotoxicity, NVP was kept in the HAART regimen because of a transient elevation of liver function enzymes. Zidovudine was switched to stavudine owing to grade 3/4 anaemia (n = 15), neutropenia (n = 7), and headache (n = 1). The SAEs resolved after interruption of co-trimoxazole for two cases of anaemia and 53 cases of neutropenia. Stavudine was switched to abacavir for one case of neuropathy.

Severe adverse events were reported in eight of 16 women who died (Table 2). One death attributed to severe anaemia may have been drug-related or exacerbated but further details around the cause of death were not available. For one woman, the causes of death were unknown. Zidovudine was switched to stavudine 9 months after HAART initiation because of a persistent severe neutropenia. About 16 months initiating stavudine, she presented myalgia and received an analgesic. An appointment was given to her 10 days later but she did not come. She died at home one month later.

### Factors associated with the of severe adverse events

In a multivariate Cox regression analysis, none of the factors investigated (age, previous exposure to PMTCT regimen, BMI, hemoglobin and neutrophil at baseline, and HAART initiation during pregnancy) was statistically associated with the first occurrence incidence of SAEs (n = 88) after adjusting for CD4 cell count and WHO clinical stage at enrollment (Table 3). When restricting the analyses to severe hepatotoxicity, only ALT ≥31 UI/L was associated with these SAEs (Table 3). The result did not change when using combined outcomes: rash or severe hepatotoxicity (data not shown). Similarly, when restricting the analyses to anaemia and/or neutropenia, only low baseline neutrophil count <1500/mm^3 ^was associated with these SAEs (Table 3). Analysis with on treatment approach led to similar results (data not shown).

**Table 3:** Factors associated with severe adverse events in women initiating NVP-based antiretroviral therapy.

	All severe adverse events (N = 88)†			Severe hepatotoxicity(N = 10)			ZDV-related severe averse events (N = 71)		
	
	HR	95% CI	p	HR	95% CI	p	HR	95% CI	p
**Age (years)**									
≥30 (n = 137)	1.03	0.67-1.59	0.90	1.37	0.37-5.06	0.64	0.92	0.57-1.49	0.74
<30 (n = 153)	1.00	-	-	1.00	-	-	1.00	-	-
**Body mass index (Kg/m^2^) **									
<18 (n = 40)	1.08	0.53-2.20	0.05				1.12	0.52-2.42	0.78
≥18 (n = 160)	1.00	-	-				1.00	-	-
**CD4+ count/mm **^**3**^									
>250 (n = 88)	0.68	0.41-1.12	0.14	1.75	0.48-6.39	0.40	0.64	0.36-1.14	0.13
≤250 (n = 202)	1.00	-	-	1.00	-	-	1.00	-	-
**WHO staging **									
Stage 3 or 4 (n = 130)	1.00	0.63-1.58	0.99	1.31	0.36-4.74	0.68	1.07	0.65-1.79	0.77
Stage 1 or 2 (n = 160)	1.00	-	-	1.00	-	-	1.00	-	-
**ALT (IU/L) **									
≥31(n = 50)	1.58	0.89-2.78	0.12	7.09	1.59-31.62	**0.01 **	1.10	0.57-2.15	0.78
<31(n = 240)	1.00	-	-	1.00	-	-	1.00	-	-
**Neutrophil level (/mm**^**3**^**) **									
<1500 (n = 28)	1.51	0.76-2.98	0.24				2.20	1.09-4.43	**0.03 **
≥1500 (n = 262)	1.00	-	-				1.00	-	-
**Hemoglobin level (g/dL) **									
≤9.8 (n = 150)	1.15	0.73-1.82	0.56	0.66	0.16-2.79	0.57	1.35	0.81-2.23	0.25
>9.8 (n = 140)	1.00	-	-	1.00	-	-	1.00	-	-
**Status of pregnancy **									
Pregnant (n = 125)	0.98	0.60-1.64	0.98	1.22	0.22-6.62	0.82	1.11	0.65-1.91	0.70
Non pregnant (n = 165)	1.00	-	-	1.00	-	-	1.00	-	-

Multivariable Cox regression analyses. MTCT-Plus program, Abidjan, Côte d'Ivoire (2003-2006).

HAART: Highly active antiretroviral therapy; WHO: World Health Organization; IQR: interquartile range; NVP: nevirapine; ALT: alanine aminotransferase; OR: odds ratio; aOR: adjusted odds ratio; CI: confidence interval, † If a woman had multiple severe adverse event

## Discussion

In Abidjan, Côte d'Ivoire, we followed 290 HIV-infected women who started ZDV/3TC/NVP in combination with co-trimoxazole. During a median follow-up on HAART of 25 months, we observed a relatively high incidence rate of SAEs of 19.5/100 patient-years. The risk factors identified for developing a SAE were elevated baseline transaminase levels for the occurrence of a rash and/or hepatotoxicity and low baseline neutrophil count for anaemia and/or neutropenia.

This high incidence rate of SAEs observed in our study was due to the high incidence of grade ¾ neutropenia (13.3/100 PY), probably due to the association ZDV and co-trimoxazole. Approximately 90% (53/60) of grade ¾ neutropenia disappeared after the interruption of co-trimoxazole and only seven women had to stop ZDV owing to neutropenia. This result is consistent with a study conducted in Côte d'Ivoire where the 118 patients with incident grade ¾ neutropenia were all receiving co-trimoxazole when the neutropenia was detected. After the interruption of co-trimaxazole, 73% of these SAEs disappeared [21]. The incidence rate of anaemia estimated in our study is also consistent with this study conducted in Côte d'Ivoire and other studies in Africa [21-23].

Concerning cutaneous rash or hepatotoxicity, most reports have expressed the risk of SAEs using a cruder indicator, the cumulative frequency. We estimated that the cumulative frequencies of grade ¾ cutaneous rash and hepatotoxicity were 5.2% et 3.4%, respectively. This result is consistent with other studies realized in resource-limited settings which have found frequencies of grade ¾ rash ranging between 2.4% and 4.6% [13-16,24] and grade ¾ hepatotoxicity ranging between 1.5% and 6.6% [12-16]. The frequency of severe hepatotoxicity is lower in resource-limited settings than in developed countries where this frequency is between 5.8% and 17.6% [7,9,25-27]. This difference could be explained by a different susceptibility to NVP between people from resource-limited settings and Caucasian origin [28], a different distribution of the frequency of some risk factors such as hepatitis B and C [27], drug use and alcohol consumption. However, in the studies from resource-limited settings, the frequency of these risk factors has not been estimated. The other risks factors associated with the hepatotoxicity and/or rash reported in litterature, such as baseline HIV-1 RNA level, NVP plasma concentration, and genetic factors, could not be investigated in our study because there are not recorded in routine circumstances in Côte d'Ivoire.

In our cohort, a CD4 count >250 cells/mm^3 ^was not associated with occurrence of SAEs, including severe hepatotoxicity and/or cutaneous rash, as was the case in most studies conducted in resource-limited-settings [13-16]. One study conducted in Mozambique among 146 pregnant women found a higher rate of severe hepatotoxicity in women with CD4 >250 cells/mm^3 ^(6.0% vs 0.0%; p = 0.02) [12]. Generally in resource-limited-settings, a great majority of HIV-infected patients initiate their treatment late, generally when the CD4 cell count is below 200 cells/mm^3 ^[29,30]. In our study, the median CD4 cell count was 188 cells/mm^3 ^and only a few women (30%) had a CD4 cell count above 250 cells/mm^3^. Moreover in this cohort, 47% of the women who had a CD4 cell count ≤250 cells/mm^3 ^initiated HAART during pregnancy, which may have resulted in an underestimation of the CD4 count values due to physiological hemodilution [31]. We also found that the initiation of HAART during pregnancy was not associated with the occurrence of severe rash or hepatotoxicity. We observed that the median duration of follow-up of pregnant women was relatively short (three months) but covered the higher-risk period for occurrence of rash or hepatotoxicity.

When investigating the causes of death, we found that half of these women (eight) had a history of SAEs during their follow up and two cases can be associated with medication toxicity: the woman who died after presenting grade 4 anaemia and those who died after presenting myalgia. This could be due to lactic acidosis. For the other deaths, it is difficult to attribute them to adverse events because of the delay between the history of SAEs and the time of death as well as the fact that documenting cause of death in these settings is a real challenge. Further pharmacovigilance studies should be conducted to document the cause of death in relation to SAEs.

This study had two main limitations. First, the study sample size was relatively small. This could reduce the power to detect difference between the risk factor groups and so the results should be interpreted with caution. However, our results provided additional information on the relation between CD4 counts >250 cells/mm^3^, pregnancy and severe adverse events. Few previous studies in sub-Saharan Africa evaluated this association and did not find any difference [13-16]. Second, we did not include data on hepatitis B virus, hepatitis C, alcohol consumption and drug use which are not routinely collected in Cote d'Ivoire's national HIV program.

Overall, data reported in this MTCT-Plus cohort is reassuring about the safety of NVP-based HAART in routine circumstances. We did not observe severe events such as Stevens-Johnson syndrome or Lyell syndrome, and almost all the SAEs disappeared after discontinuation of the incriminated drug.

## Conclusion

This prospective cohort study provides additional data in West Africa where CRF02 is the predominant of HIV-1 subtype and where the population was different. As shown by almost all studies conducted in resource-limited settings, including our study, a high absolute CD4 cell count was not associated with a higher risk of severe hepatotoxicity and/or rash, as well as pregnancy. However, as the sample size of these studies was limited, these results should be interpreted with caution. Data on women receiving NVP in resource-limited settings remain limited and there is a pressing need for better information about NVP toxicity in these settings. We believe that further studies especially meta-analyze with data available in low-income countries are urgently needed. NVP-based HAART should continue to be used with caution and close monitoring in eligible women for HAART with absolute CD4 cell count between 250 and 350 cells/mm^3 ^and in eligible pregnant women in resource-limited settings if no other options exist.

## Competing interests

The authors declare that they have no competing interests.

## Authors' contributions

PAC, BTG, AKT, CAB, GB, EJA, FD and DKE agree with the manuscript's results and conclusions. PAC and DKE designed the study, PAC, CAB, GB collected the data. PAC and DKE analyzed and interpreted the data: PAC, BTG, AKT, CAB, GB, EJA, FD and DKE contributed to the writing of the manuscript. There is no conflict interest to declare. All authors read and approved the final manuscript.

## Pre-publication history

The pre-publication history for this paper can be accessed here:

http://www.biomedcentral.com/1471-2334/10/188/prepub
